# State of the *JACMP*, 2023

**DOI:** 10.1002/acm2.13921

**Published:** 2023-01-31

**Authors:** Michael D. Mills

**Affiliations:** ^1^ Department of Radiation Oncology University of Louisville Louisville Kentucky USA

There were more than a few changes to the *JACMP* in late 2022 and early 2023. Elle Thomas, the Medical Physics Managing Editor joined the *JACMP* also as Managing Editor in October, 2022. She is performing a management supporting role for the *JACMP* and brings significant knowledge and skills to the *JACMP* operation. The *JACMP* and *Medical Physics* utilize the same publisher and the same manuscript management platform, Wiley and eJournalPress respectively, which mean that a lot of the operational processes are the same for both journals. All members of the Editor‐in‐Chief team are elated that Elle was chosen to work with the *JACMP*.

A second change is that Per Halvorsen has accepted a position that will greatly limit his continuing as Deputy Editor‐in‐Chief. Per completed his term of service at the end of 2022, but will remain on the *JACMP* as Deputy Editor‐in‐Chief Emeritus and will voluntarily edit a very limited number of manuscripts, primarily in the category of AAPM Reports and Documents. Per has served the *JACMP* as Deputy Editor‐in‐Chief since January of 2013 and has performed extraordinary service over the years. We will miss him but also, we are thankful to have him in this continuing role as occasionally the EIC team is faced with difficult decisions. It is helpful to have his wise and seasoned perspective and advice when important decisions are made.

With Per's departure, the *JACMP* had an opening for a new Deputy Editor‐in‐Chief for the *JACMP*. After a search limited to those who have demonstrated noteworthy contributions to the *JACMP*, the consensus choice was that Susan Richardson join the Editor‐in‐Chief team. Susan is a former member of the AAPM EXCOM having served the AAPM as Secretary for 3 years. She is an expert in clinical brachytherapy, medical physics education, and AAPM operations. Additionally, she is a careful and skilled writer, scholar and editor. It is a great pleasure for Tim Solberg and me to welcome Susan to the EIC team.

Finally, the *JACMP* added a number of new Associate Editors in January, 2023. These AEs were identified from the most productive, competent and efficient *JACMP* reviewers. The EIC team welcomes each of these new AEs to the *JACMP* Board of Editors.

We are seeing continued improvement in certain critical *JACMP* benchmarks. The average number of days from acceptance of an article to publication has decreased from 47 days in 2017 to 34.5 days in 2022 The Impact Factor for the *JACMP* was 1.118 in 2013, 1.301 in 2018, and 2.243 in 2022. Articles published totaled 329, 349, and 338 in 2020, 2021, and 2022, respectively. The 2022 *JACMP* acceptance percentage was 56%. There were 152.5 days from submission to publication in 2022 compared to 163 days in 2021. There were 12,062, 16,502, and 20,376 registered *JACMP* users at the end of 2020, 2021, and 2022, respectively.

The clinical emphasis of the *JACMP* illustrates why the AAPM needs two scholarly journals. However, there is some overlap between the two journals; about 1/3 of the articles published in the *JACMP* might also have been included in the *Medical Physics* publication space. For these articles, the venue of publication is mostly the decision of the authors. For those submitted articles that are not a proper fit for the journal selected by the author, Wiley has put in place a streamlined method to transfer articles immediately and seamlessly from one journal to the other. Note that the journal Editorial Boards differ in makeup; *JACMP* Associate Editors and reviewers tend to be clinical while *Medical Physics* editors and reviewers are mostly focused toward scientific contributions.

Finally, I ask all members of the *JACMP* community to take a few moments and help us by logging in to your profile to complete your areas of expertise. Many thanks for doing this. The instructions are below:

***JACMP*—Modify Areas of Expertise**

Please follow these steps below to login, access the profile screen, and modify the profile screen.

1. Login—https://jacmp.msubmit.net/cgi‐bin/main.plex
  - If you have forgotten your Login Name, it is best to send an email to JACMPEditorial@wiley.com
  - If you have forgotten your password, click on “Forgot your password?” just above the Login and Reset buttons on the login page.
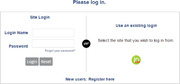

*New users will be prompted to fill out affiliation/institution information as well as specifying areas of expertise before the registration process is completed*.

1. After logging in the site is separated into several subsections. In the bottom‐most subsection, entitled General Tasks, click on “Modify Profile/Password”
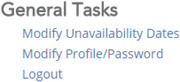
2. This is the modify profile page. Here you are able to amend nearly every aspect of your profile and user settings. Scroll down to the bottom of the modify profile page to until you see “Will you consider being a Reviewer for this journal?”
  - Select by clicking on the circle to the left of your answer.
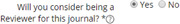
3. After the reviewer question, you will see two sections for “Areas of Expertise.” The first section you can search by two methods.
  - Type key words into the “Search Areas of Expertise” text box.
  - Click on “I,” “M,” “T” to manually search the taxonomy.
  - When you find the area you wish to choose, simply click on the text. The area you clicked on will be transferred to the box on the right (your selected areas).
4. The second section is used for manually entering keywords you cannot find in the first section. Both sections are used by our database when Associate Editors search for potential reviewers.
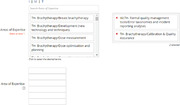

After the fields are modified click on “Modify Profile / Continue” at the bottom of the page to complete the amendments. This is also a good time to update your institutional information and link your existing ORCID profile. Any questions, concerns, or errors while modifying your profile should be sent to JACMPEditorial@wiley.com.

